# A Digital Innovation for Enhanced Adherence Counseling (EAC) to Improve Outcomes Among People Living With HIV (PLHIV) in Mumbai, India

**DOI:** 10.7759/cureus.100601

**Published:** 2026-01-02

**Authors:** Shrikala Acharya, Vijay Kumar Karanjkar, Mohit Goyal, Amol Palkar, Prashant Deshpande, Sachin Dhande, Angom Peter Singh, Dhirubhai Rathod, Maninder S Setia

**Affiliations:** 1 Community Medicine, Lokmanya Tilak Municipal Medical College and General Hospital, Mumbai, IND; 2 Epidemiology and Public Health, Mumbai District AIDS Control Society, Mumbai, IND; 3 Epidemiology and Public Health, International Training and Education Center for Health (I-TECH), New Delhi, IND; 4 Data Management, International Training and Education Center for Health (I-TECH), New Delhi, IND; 5 Epidemiology and Public Health, MGM Institute of Health Sciences, Navi Mumbai, IND

**Keywords:** adherence, digital application, enhanced adherence counselling, programmatic data, viral load

## Abstract

Introduction

Early initiation of antiretroviral therapy (ART) and adequate adherence are associated with sustained viral load (VL) suppression and effective treatment in people living with HIV (PLHIV). Enhanced adherence counseling (EAC) has been recommended for PLHIV on therapy with a VL >1000 copies/mL. We developed a digital application, "Samvaad," for counselors at ART centers to document the barriers to poor adherence and to provide thematic EAC for registered PLHIV under the aegis of the Mumbai District AIDS Control Society. The objectives of this study are to document the barriers to ART adherence in PLHIV who require EAC and to report the suppression outcomes in those who received EAC using Samvaad.

Methods

This study is a pre-post analysis of retrospective programmatic data from 674 PLHIV across 16 ART centers in Mumbai, India, from September 2020 to July 2022. We included only PLHIV who had unsuppressed VL and/or ART adherence <95% for the present analysis. The main outcome was the change in VL status, from unsuppressed at baseline to suppressed at follow-up assessment. We collected demographic information, ART-related information (duration of ART and type of ART regimen), and CD4 counts at the time of EAC. We also documented barriers to ART adherence using "Samvaad."

Results

The mean (SD) age of PLHIV was 37.1 (9.9) years; 60.7% (n=409) were male, and 39.3% (n=265) were female. The most common barrier at baseline was "I have not been adequately informed about the dose and schedule of medications" (65.1% (n=439)), followed by "I do not have a fixed time to take my medicines" (63.2% (n=426)). The least common barriers were "I skip medications in the morning whenever I have alcohol the previous night" (4.2% (n=28)), "I skip medications whenever I have a fight with my partner/lover" (4.5% (n=30)), and "I live with a lot of people and hence I am unable to keep the ART medications at home" (4.5% (n=30)). The most common domains were "pill-taking practices" (68.3% (n=460)) and "ART knowledge/behavior" (67.7% (n=456)). At follow-up assessment, about 90% of PLHIV had suppressed VLs. In the multivariate analysis, males were significantly more likely to be virally suppressed compared with females (OR: 1.99, 95% CI: 1.07-3.68; p=0.029). PLHIV on third-line ART (OR: 0.30, 95% CI: 0.11-0.85; p=0.024) and those with the practices barrier domain (OR: 0.44, 95% CI: 0.22-0.90; p=0.024) were significantly less likely to achieve VL suppression at follow-up.

Conclusions

The majority of PLHIV in our study were between 26 and 45 years of age, were male, had been on ART for more than five years, and were on first-line ART. The main barriers were a lack of adequate knowledge about dosage and side effects, as well as not having a fixed schedule for taking pills. Viral suppression was reported in approximately 90% of PLHIV after EAC sessions. However, VL suppression was less likely in those who did not have a fixed time for taking medicines. This is a practical problem that needs to be addressed by developing treatment plans that consider time spent away from home or in transit. The development of a digital app was useful to document key barriers and domains in PLHIV with poor adherence and to provide thematic EAC at ART centers. The app can be used in urban as well as rural ART centers to provide client-centric thematic adherence counseling.

## Introduction

India has an estimated 2.5 million people living with HIV (PLHIV), with three states, Maharashtra, Andhra Pradesh, and Karnataka, accounting for nearly 39% of total cases in the country [1]. Maharashtra has the highest number of recorded PLHIV, and Mumbai, the capital of Maharashtra with an estimated case burden of 62,571 PLHIV, is a high-priority area for HIV prevention and care [1]. The government of India initiated a free antiretroviral therapy (ART) program in April 2004 through eight ART centers, which has now been expanded nationwide with a "test and treat" policy [2]. India is committed to achieving the UNAIDS (Joint United Nations Programme on HIV/AIDS) target of 95-95-95 [3-5]. Recent statistics show that 79% of PLHIV in India are aware of their status, 86% are on ART, and 93% have suppressed viral loads (VLs) [6]. Early initiation of ART and adequate adherence to treatment are crucial for sustained VL suppression and effective treatment in PLHIV [7]. The World Health Organization has recommended that VL be used for diagnosing and confirming treatment failure. Additionally, regular monitoring of VL may be used to provide feedback to PLHIV on their treatment and the importance of adherence [8].

Poor adherence to ART may be due to side effects of medications, family and social obligations, stigma, nondisclosure to family members, lack of adequate knowledge, alcohol use, forgetting to take pills, and mental health issues [9-15]. Counseling about ART use is a standard part of managing PLHIV, but enhanced adherence counseling (EAC) has been recommended for PLHIV with a VL >1000 copies/mL [16-19]. The sessions are to be conducted over a period of three to six months with a repeat VL test at the end [16]. In India, HIV treatment guidelines recommend "step-up adherence counseling" for PLHIV with suspected treatment failures [2]. These counseling sessions should be conducted over three sessions and include detailed discussions about VL interpretation, barriers to adherence, medical history, and referral systems. Counselors should provide strategies for overcoming these barriers, explore support systems, offer referral services as needed, reassess VL after three months of good adherence, and consider changing the regimen if VL is still not suppressed [2].

EAC has been conducted in various settings using multiple approaches. Some studies have used physical counseling sessions, while others have utilized phone-based EAC sessions [20-23]. Ekejiuba and colleagues studied both phone-based and physical EAC and found no significant differences in VL suppression after EAC between these groups [24]. Another study used smartphone-based strategies to promote adherence to ART, but they also did not find any significant differences in the overall adherence between the smartphone strategy group and the control group over time [25]. The premise of EAC is to offer person-centered solutions to overcome barriers. This person-centered counseling is possible within the context of continuity with the same counselor at every visit and access to previous records with the healthcare provider/counselor. The lack of tools to document the EAC process often leads to incomplete documentation, and the unavailability of the same counselor during follow-up visits, along with high patient loads, remains a challenge at ART centers in providing person-centric EAC within programmatic settings.

To address some of these concerns, we developed a digital application, "Samvaad," for counselors at ART centers in Mumbai to document barriers to treatment adherence and provide prompts for EAC for PLHIV registered at ART centers under the Mumbai District AIDS Control Society (MDACS). This was done as part of service delivery in the HIV program in Mumbai, India. The objectives of this study are to document the barriers to ART adherence in PLHIV who required EAC and to report the suppression outcomes in those who received EAC using "Samvaad."

## Materials and methods

This study is a pre-post analysis of retrospective programmatic data of 674 PLHIV from 16 ART centers during the period from September 2020 to July 2022 (initial period of the use of this application in the public health program) in Mumbai, India.

Study site and population

**Figure 1:**
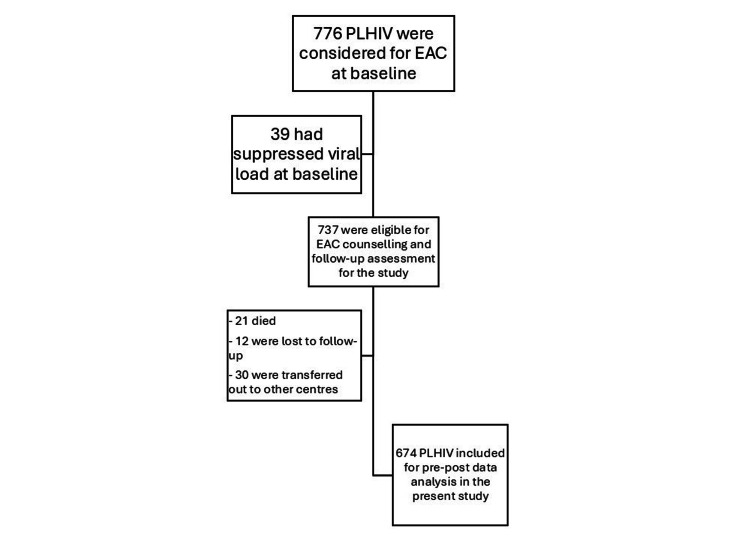
Flowchart of PLHIV inclusion in the study, Mumbai, India. PLHIV, people living with HIV; EAC, enhanced adherence counseling

MDACS provides HIV-related comprehensive care, support, and treatment services to PLHIV in Mumbai through ART centers. In these 16 ART centers, 776 adult PLHIV (≥18 years) were considered for EAC. We included only those PLHIV who had unsuppressed VLs and/or ART adherence <95% for the present analysis. Treatment adherence among PLHIV enrolled in care is evaluated by the ART counselor at each visit to the ART center. This assessment is conducted using a checklist that reviews any missed doses since the previous visit, along with a pill count of the remaining medication brought by the client. Adherence is then calculated as a percentage by comparing the number of pills consumed with the number expected to be taken during the specified period and is recorded on the treatment card. Of these 776 PLHIV, 39 had suppressed VL at baseline, so they were excluded from the present analysis. Among the 737 included PLHIV, 21 died, 12 were lost to follow-up, and 30 were transferred to other centers during the period. Thus, for the final pre-post analysis, we included data from 674 PLHIV (Figure 1).

Development of the Samvaad application and enhanced adherence counseling process

**Figure 2:**
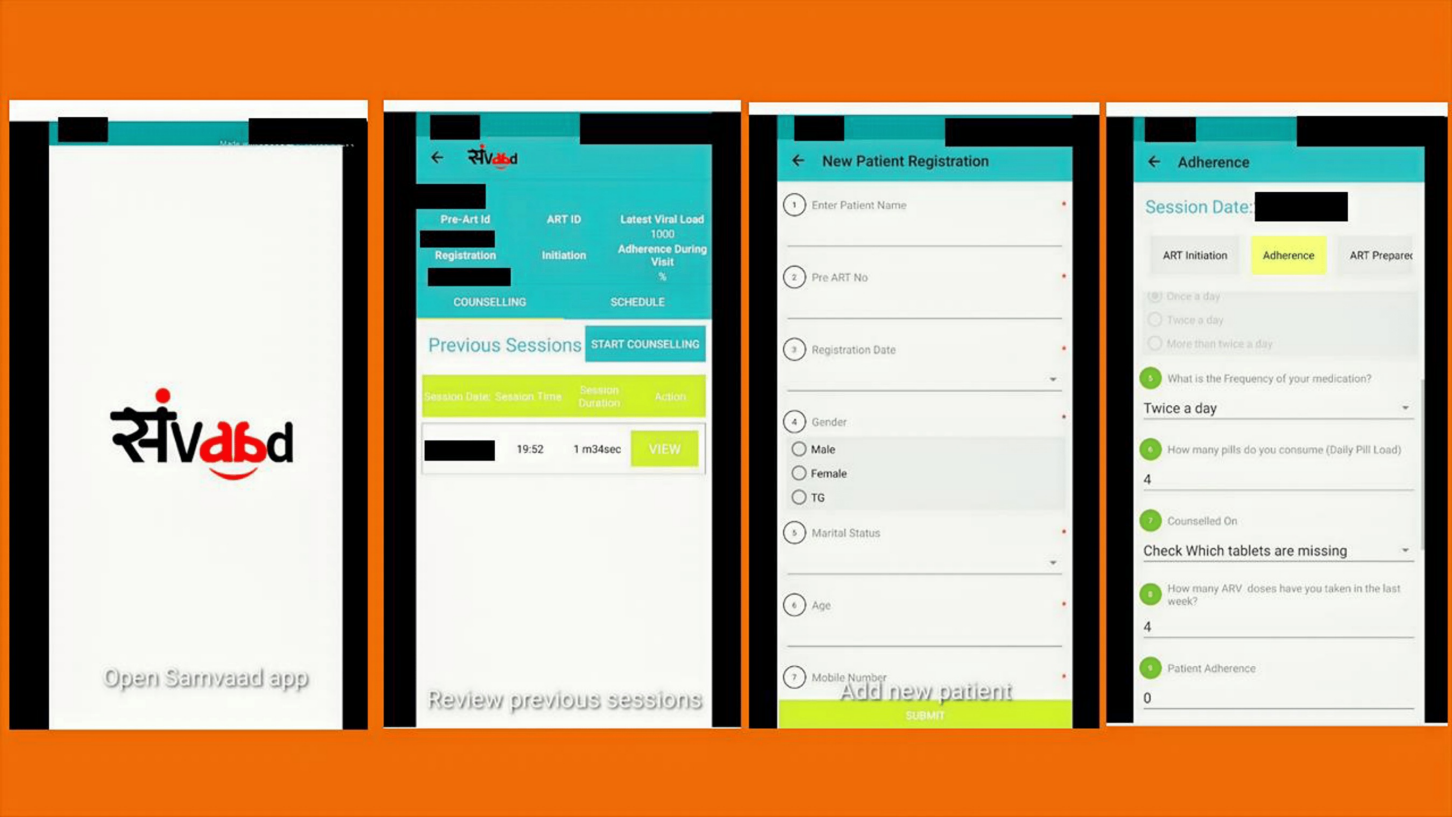
Selected aspects of the "Samvaad" application. The screenshots were taken from the application, and identifiers have been masked (black cover).

We developed a digital application named "Samvaad" (a Hindi word meaning "dialogue") to enhance the quality of interactions between counselors and clients (PLHIV) at ART centers as part of the HIV service program in Mumbai. This digital application, Samvaad, facilitated structured counseling through eight thematic modules: 1) ART preparedness and initiation; 2) disclosure; 3) adherence to ART and EAC; 4) sexual and reproductive health; 5) missed appointments; 6) alcohol use; 7) mental health issues; and 8) multi-month dispensation of ART. In the ART adherence and EAC module, the application could identify potential barriers in these domains: 1) ART knowledge and behavior; 2) stigma; 3) perception about ART; 4) pill-taking practices; 5) mental health and stress; and 6) substance use. The app was developed using an algorithm that prompted the appropriate counseling module to be used based on the barriers recorded in the app. Additionally, the app provided pointers on the topics to be covered in the counseling session. We had initially conducted a baseline study, and these domains and barriers within each domain were identified from this analysis [26]. In addition, we conducted group discussions with PLHIV to discuss these barriers and their use in the app. We also conducted group discussions with counselors at ART centers and consultations with HIV experts and counselors who are not part of these centers regarding the suitability and applicability of the app. The counselors in the ART centers were trained in motivational interviewing techniques to identify challenges, document key barriers to adherence, deliver personalized solutions, and track progress using the Samvaad app. Each counselor had a user ID to access the Samvaad application on smartphones or tablets, and the information of PLHIV clients was stored using unique identifiers to track progress. The data of individual PLHIV were imported from a master list, and during the session, the application dynamically identified the relevant module based on the PLHIV characteristics. The program was coded to prompt key counseling strategies based on the reported barriers and domains. Counselors recorded the barriers, provided thematic adherence counseling, and revisited progress in subsequent sessions, creating a structured approach to resolve the barriers (Figure 2). This was implemented for all PLHIV registered for care in all ART centers in the city.

Study variables

The main outcome was a change in VL status, from unsuppressed at baseline to suppressed at follow-up assessment. We collected demographic information (age, gender, and marital status), ART-related information (duration of ART and type of ART regimen), and CD4 counts at the time of EAC. We also recorded the barriers (of the 13 barriers included in the app) reported by PLHIV. These barriers were included in six barrier domains (knowledge/behavior about ART, perception about ART, pill-taking practices, stigma related to ART, stress/mental health issues, and substance use). We collected information on the number of EAC sessions for each PLHA. Finally, we recorded the VL and ART adherence after the last EAC session. The last VL count was used to calculate the outcome (unsuppressed at baseline and suppressed at follow-up).

Statistical methods

The primary outcome for the present analysis was suppressed VL at follow-up after EAC sessions. We estimated the means and SD or median and IQR for linear variables and proportions for categorical variables. We used the chi-square test to compare the proportion of outcomes across demographic and ART-related characteristics. The proportions were also compared across various domains and the number of EAC sessions. The proportion over ordinal categories was also assessed using the chi-square for trend. We used logistic regression models to estimate OR and their 95% CI. We initially built models with individual variables for unadjusted estimates and then used the multivariate regression models for adjusted OR. The fit of the model was assessed using the Akaike Information Criteria and Bayesian Information Criteria, and variance inflation factors were used for the models to avoid multicollinearity. We used methods described by Vittinghoff and colleagues to assess the trend in logistic regression models [27]. A p-value <0.05 was considered statistically significant.

Data were analyzed using Stata version 17 (© StataCorp, College Station, Texas, USA).

Ethics approval

The study was approved by the MDACS Ethics Committee on October 14, 2022 (reference number: MDACS/IRB 3275A). The study was conducted in accordance with the principles of the Declaration of Helsinki and Good Clinical Practices. The study used retrospective data; hence, a waiver of consent was sought for the analysis of data and was granted by the Ethics Committee.

## Results

The mean (SD) age of PLHIV was 37.1 (9.9) years; 60.7% (n=409) were male, and 39.3% (n=265) were female. The mean age was significantly higher in males compared with females (38.2 (9.9) vs. 35.3 (9.6); p=0.0002). The majority of PLHIV (58.8% (n=396)) were married or living with a partner, 25.9% (n=175) were separated or their partner had died, and 15.3% (n=103) were single. The median (IQR) CD4 cell count before the EAC session was 451 (293, 639) cells/mm³. The majority of these patients had been on ART (57.7% (n=389)) for more than five years and were on a first-line ART (50.3% (n=339)) regimen. Additional demographic and clinical details of PLHIV who underwent EAC are presented in Table 1.

**Table 1:** Baseline demographic and clinical characteristics of PLHIV eligible for EAC in Mumbai. Data are presented as n and percentage; these are column percentages with 674 as the base. Linear data are presented as median and IQR. EAC, enhanced adherence counseling; PLHIV, people living with HIV; ART, antiretroviral therapy

Characteristics	n (%)
Total	674 (100)
Age group (in years)	
19-25	95 (14.1)
26-35	218 (32.3)
36-45	226 (33.5)
≥46	135 (20.0)
Median age in years (IQR)	37 (30, 44)
Gender	
Male	409 (60.7)
Female	265 (39.3)
Marital status	
Single	103 (15.3)
Married/living with partner	396 (58.8)
Separated/widowed	175 (25.9)
CD4 count (cells/mm^3^) at enrollment into EAC	
0-100	17 (2.5)
101-200	67 (9.9)
201-350	136 (20.2)
351-500	179 (26.6)
≥501	275 (40.8)
Median CD4 cells/mm^3^ (IQR)	451 (293, 639)
Duration on ART (in months)	
0-24	88 (13.1)
25-60	197 (29.2)
61-120	295 (43.8)
>120	94 (13.9)
Median duration on ART (IQR)	71 (39, 102)
Type of treatment	
1st line	339 (50.3)
2nd line	296 (43.9)
3rd line	39 (5.8)

**Figure 3:**
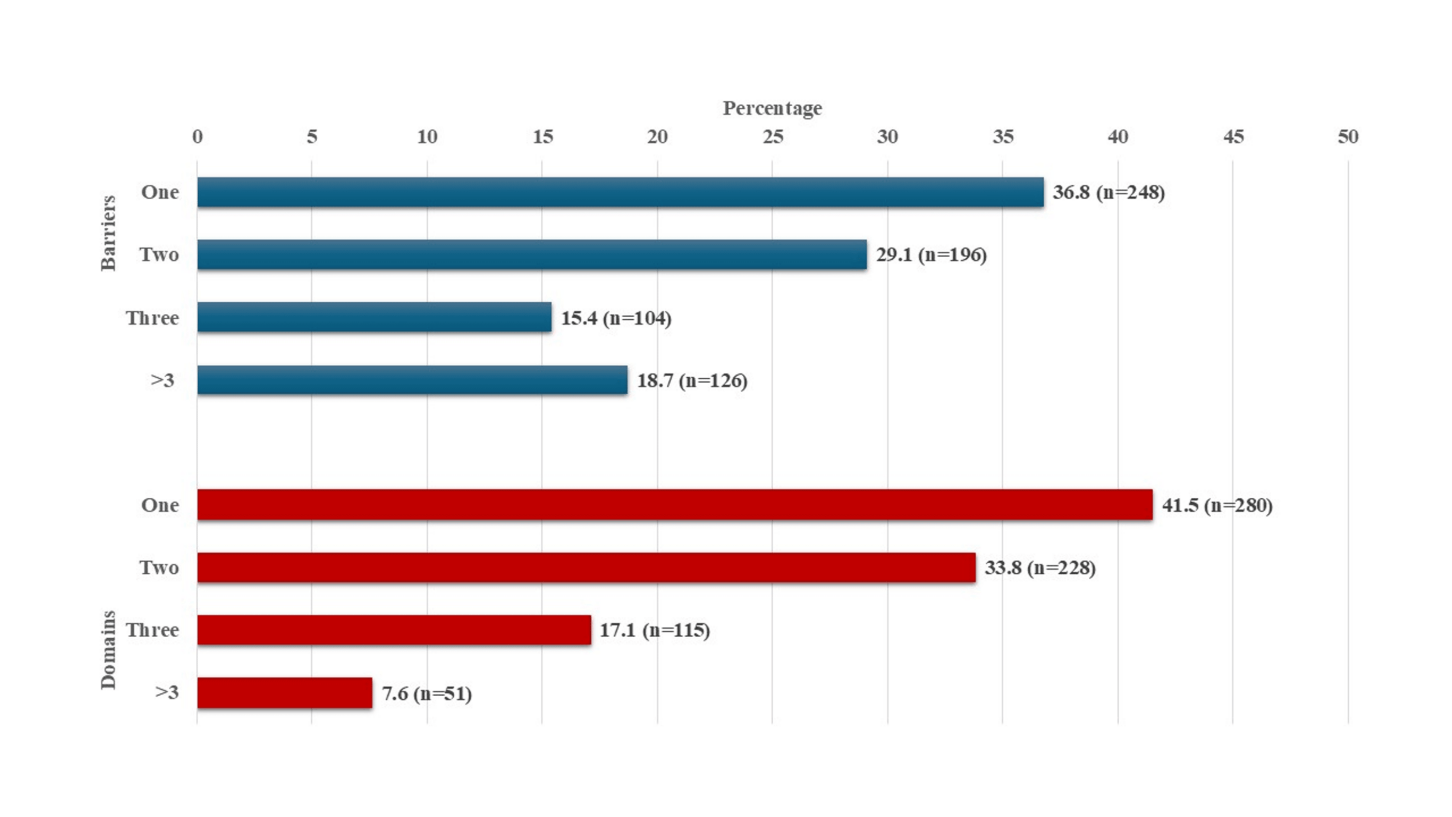
Number of domains and barriers for ART adherence among 674 PLHIV in Mumbai, India. Bars represent percentages, with "n" provided in parentheses. PLHIV, people living with HIV; ART, antiretroviral therapy

The most common barrier at baseline was "I have not been adequately informed about the dose and schedule of medications," as reported by 65.1% (n=439) of PLHIV, followed by "I do not have a fixed time to take my medicines," reported by 63.2% (n=426). The least common barriers were "I skip medications in the morning whenever I have alcohol the previous night" (4.2% (n=28)), "I skip medications whenever I have a fight with my partner/lover" (4.5% (n=30)), and "I live with a lot of people and hence I am unable to keep the ART medications at home" (4.5% (n=30)). The most common domains were "pill-taking practices" (68.3% (n=460)) and "ART knowledge/behavior" (67.7% (n=456)). We have presented a detailed analysis of barriers and domains in Table 2. Most of the PLHIV reported only one (36.8% (n=248)) or two barriers (29.1% (n=196)), and 41.5% (n=280) could be categorized into a single domain (Figure 3). The median (IQR) number of barriers in our population was 2 (1, 3). The majority of them received one session of EAC (67.8% (n=457)), and 16.6% (n=112) had received two sessions.

**Table 2:** Barriers and domains for ART adherence among PLHIV receiving EAC in Mumbai, India (n=674). Data are presented as n and percentage for each barrier and as the sum of barriers for each domain. These are column percentages with 674 as the base. EAC, enhanced adherence counseling; PLHIV, people living with HIV; ART, antiretroviral therapy

Domain	Barrier statement	Proportion for each barrier	Proportion for each domain
		N (%)	N (%)
ART knowledge/behavior	I have not been informed adequately about the dose and schedule of medications	439 (65.1)	456 (67.7)
	I avoid taking medications because of side effects	62 (9.2)	
ART stigma	I skip taking medicines whenever people are around	75 (11.1)	108 (16.0)
	I live with a lot of people and hence I am unable to keep the ART medications at home	30 (4.5)	
	I may lose my business/job if others come to know that I am HIV positive through me taking ART/attending ART center	39 (5.8)	
Perception about ART	I avoid taking medications whenever I do not have enough food to eat	147 (21.8)	183 (27.2)
	I believe that the medicines do more harm than good to me	112 (16.6)	
Pill-taking practices	I do not have a fixed time to take my medicine	426 (63.2)	460 (68.3)
	It can be very difficult to take medications daily	150 (22.3)	
Stress/mental health	I skip medications whenever I feel low	50 (7.4)	68 (10.1)
	I skip medications whenever I have a fight with my partner/lover	30 (4.5)	
Substance use	I skip medication after taking recreational drugs	21 (3.1)	41 (6.1)
	I skip medications in the morning whenever I have alcohol the previous night	28 (4.2)	

We also compared the barriers between male and female PLHIV. Female PLHIV were significantly more likely to report "I avoid taking medications because of side effects" (13.2% vs. 6.6%; p=0.004) and "I skip medications whenever I have a fight with my partner/lover" (6.4% vs. 3.2%; p=0.047) compared with males. However, male PLHIV were more likely to report "I skip medications in the morning whenever I have alcohol the previous night" compared with female PLHIV (5.4% vs. 2.3%; p=0.048). There was no significant difference for other barriers or domains.

In the follow-up assessment, about 89.5% (n=603) of PLHIV had suppressed VLs. We found that younger PLHIV were significantly more likely to have unsuppressed VLs at follow-up; the chi-square for trend was also statistically significant for age groups (Table 3). Gender and marital status were not associated with VL suppression after EAC sessions. However, ART duration and CD4 cell counts were significantly associated with VL suppression. As seen in Table 3, PLHIV who were on ART for less than two years and those with a CD4 count up to 200 cells/mm³ were significantly less likely to have suppressed VL in the follow-up assessment. There was no significant association between the type of ART regimen and VL suppression at follow-up. However, PLHIV who reported barriers in the "practices" and "mental health" domains were significantly less likely to have VL suppression at follow-up. In addition, PLHIV classified into more than one barrier domain were significantly less likely to have VL suppression. However, the number of EAC sessions was not associated with VL suppression at the follow-up assessment. Detailed proportions and p-values are presented in Table 3.

**Table 3:** Factors associated with VL suppression among 674 PLHIV receiving EAC sessions for ART adherence in Mumbai, India. Data are presented as n and percentage. Percentages in the total column are column percentages, and those in the VL not suppressed and suppressed columns are row percentages. The test statistic is chi-square (χ²). A p-value <0.05 was considered statistically significant. *p-value from the chi-square (χ²) test for trend. EAC, enhanced adherence counseling; PLHIV, people living with HIV; ART, antiretroviral therapy; VL, viral load

	Total	VL not suppressed (>1000 copies)	VL suppressed ( <1000 copies)	Test statistic	p-values
	N (%)	n (%)	n (%)		
	674 (100.0)	71 (10.5)	603 (89.5)		
Individual-level characteristics					
Age (years)					
19-25	95 (14.1)	15 (15.8)	80 (84.2)	χ²=5.88	0.12
26-35	218 (32.3)	24 (11.0)	194 (89.0)	χ² for trend=5.07	0.02*
36-45	226 (33.5)	24 (10.6)	202 (89.4)		
≥46	135 (20.0)	8 (5.9)	127 (94.1)		
Gender					
Male	409 (60.7)	37 (9.1)	372 (90.9)	χ²=2.44	0.12
Female	265 (39.3)	34 (12.8)	231 (87.2)		
Marital status					
Single	103 (15.3)	17 (16.5)	86 (83.5)	χ²=4.62	0.099
Married/living with partner	396 (58.8)	37 (9.3)	359 (90.7)		
Partner died/separated	175 (25.9)	17 (9.7)	158 (90.3)		
ART duration (yrs)					
Up to 2	88 (13.1)	16 (18.2)	72 (81.8)	χ²=10.47	0.02
>2-5	197 (29.2)	25 (12.7)	172 (87.3)	χ² for trend=10.28	0.001*
>5-10	295 (43.8)	25 (8.5)	270 (91.5)		
>10	94 (13.9)	5 (5.3)	89 (94.7)		
CD4 group (baseline)					
0-200	84 (12.5)	18 (21.4)	66 (78.6)	χ²=15.82	0.001
201-350	136 (20.2)	17 (12.5)	119 (87.5)	χ² of trend=14.52	<0.001*
351-500	179 (26.6)	18 (10.1)	161 (89.9)		
>500	275 (40.8)	18 (6.6)	257 (93.5)		
ART regiment					
1st line ART	339 (50.3)	29 (8.6)	310 (91.4)	χ²=4.21	0.12
2nd line ART	296 (43.9)	35 (11.8)	261 (88.2)		
3rd line ART	39 (5.8)	7 (17.9)	82 (82.1)		
Domains					
Knowledge	456 (67.7)	44 (9.7)	412 (90.3)	χ²=1.17	0.28
Stigma	108 (16.0)	13 (12.0)	95 (87.9)	χ²=0.31	0.58
Perception	183 (27.2)	19 (10.4)	164 (89.6)	χ²=0.01	0.94
Practices	460 (68.3)	59 (12.8)	401 (87.2)	χ²=8.08	0.004
Mental health	68 (10.1)	12 (17.6)	56 (82.4)	χ²=4.06	0.044
Substance use	41 (6.1)	7 (17.1)	34 (82.9)	χ²=1.98	0.16
Number of domains					
1	280 (41.5)	20 (7.1)	260 (92.9)	χ²=5.85	0.016
>1	394 (58.5)	51 (12.9)	343 (87.1)		
EAC sessions					
1	457 (67.8)	49 (10.7)	408 (89.3)	χ²=0.42	0.81
2	112 (16.6)	10 (8.9)	102 (91.1)		
>2	105 (15.6)	12 (11.4)	93 (88.6)		

In the unadjusted logistic regression models, age, marital status, ART duration, CD4 counts, ART regimen, and the type of barrier domain (practices and mental health) were associated with VL suppression at follow-up. In the multivariate analysis, we found that males were significantly more likely to be virally suppressed compared with females (OR: 1.99, 95% CI: 1.07-3.68; p=0.029). PLHIV who were on ART for more than five years were significantly more likely to have suppressed VL compared with those who were on ART for two years or less. Similarly, those with a baseline CD4 cell count ≥351 cells/mm³ were significantly more likely to have VL suppression at follow-up compared with those in whom the CD4 cell count was less than 200 cells/mm³. In both these variables (ART duration and CD4 counts), the test for trend was statistically significant. PLHIV who were on third-line ART were significantly less likely to have suppressed VL on follow-up compared with those who were on a first-line ART regimen (OR: 0.30, 95% CI: 0.11-0.85; p=0.024). PLHIV with the "practices" barrier domain were significantly less likely to have VL suppression after follow-up (OR: 0.44, 95% CI: 0.22-0.90; p=0.024). There was no association between the number of EAC sessions and VL suppression at the follow-up assessment. We have presented unadjusted and adjusted ORs for all variables in Table 4.

**Table 4:** Unadjusted and adjusted OR and their 95% CI from logistic regression models for VL suppression after EAC among 674 PLHIV in Mumbai, India. Data are from logistic regression models, and p-values are from the z-statistic. A p-value <0.05 was considered statistically significant. ^a^p=0.065, ^b^p=0.082, *p<0.05, **p<0.01, ***p<0.001, ^§^test for trend p<0.05 EAC, enhanced adherence counseling; PLHIV, people living with HIV; ART, antiretroviral therapy; VL, viral load

	Unadjusted models	Adjusted models
	OR (95% CI)	OR (95% CI)
Individual-level characteristics		
Age (years)		
19-25	Reference	Reference
26-35	1.52 (0.76, 3.03)	1.19 (0.52, 2.76)
36-45	1.57 (0.79, 3.16)	0.91 (0.38, 2.18)
≥46	2.98 (1.21, 7.34)*	2.12 (0.73, 6.14)
Gender		
Female	Reference	Reference
Male	1.48 (0.90, 2.42)	1.99 (1.07, 3.68)*
Marital status		
Single	Reference	Reference
Married/living with partner	1.92 (1.03, 3.57)*	1.78 (0.83, 3.83)
Partner died/separated	1.84 (0.89, 3.78)	1.80 (0.70, 4.63)
ART duration (yrs)		
Up to 2	Reference	Reference^§^
>2-5	1.53 (0.77, 3.03)	1.45 (0.70, 3.02)
>5-10	2.40 (1.22, 4.73)*	2.33 (1.12, 4.86)*
>10	3.96 (1.38, 11/32)*	3.50 (1.15, 10.67)*
CD4 group (baseline)		
0-200	Reference	Reference^§^
201-350	1.91 (0.92, 3.95)^b^	1.59 (0.73, 3.48)
351-500	2.43 (1.20, 4.98)*	2.21 (1.03, 4.75)*
>500	3.89 (1.92, 7.90)***	3.48 (1.62, 7.49)**
ART regiment		
1st line ART	Reference	Reference
2nd line ART	0.70 (0.42, 1.17)	0.63 (0.36, 1.10)
3rd line ART	0.43 (0.17, 1.05)^a^	0.30 (0.11, 0.85)*
Domains		
Knowledge	1.32 (0.80, 2.20)	1.28 (0.73, 2.24)
Stigma	0.83 (0.44, 1.58)	1.28 (0.60, 2.73)
Perception	1.02 (0.59, 1.78)	1.52 (0.80, 2.91)
Practices	0.40 (0.21, 0.77)**	0.44 (0.22, 0.90)*
Mental health	0.50 (0.26, 0.99)*	0.57 (0.25, 1.28)
Substance use	0.54 (0.23, 1.28)	0.69 (0.25, 1.89)
EAC sessions		
1	Reference	Reference
2	1.23 (0.60, 2.50)	1.13 (0.53, 2.42)
>2	0.93 (0.48, 1.82)	0.92 (0.43, 1.95)

## Discussion

The majority of PLHIV in our study were between 26 and 45 years of age, were male, had been on ART for more than five years, and were on first-line ART. The main barriers were a lack of adequate knowledge about dosage and side effects, as well as not having a fixed schedule for taking pills. The common barrier domains in our study population were "pill-taking practices" and "ART knowledge/behavior" related issues. A very high proportion of PLHIV had suppressed VLs after EAC sessions. Factors associated with the suppression of VL after EAC were male gender, longer duration of ART (>5 years), and high baseline CD4 counts. However, PLHIV on third-line ART and those who reported the "pill-taking practices" domain as a barrier were significantly less likely to have VL suppression after EAC sessions.

The two important barriers identified were a lack of adequate knowledge about medication dosages and not having a fixed time for taking medications. In a previous quantitative study with key populations (such as female sex workers and men who have sex with men), we found that common barriers included missing doses after consuming alcohol, fear of losing business if others knew about their HIV status, and treatment interrupting daily activities [26]. In fact, being inadequately informed about dosage and schedule was reported by 6%, and not having a fixed time was reported by only 10% of these individuals. Thus, it appears that there are different barriers between the general population and key populations, even within the same settings. Therefore, counselors providing adherence counseling should inquire about specific barriers and provide targeted counseling for these issues. Other authors [28] have also found a lack of adequate knowledge and an irregular schedule to be barriers to ART adherence. However, an important issue identified by them was "no medications due to lack of insurance coverage or inadequate coverage." Since ART is provided free of charge by the government of India through its ART centers, this was not identified as a barrier in our population. In another study, Genberg and colleagues found that "family responsibilities" and "stigma" were the most common barriers, while "scheduling difficulties" were the least important barrier [29]. Heylen and coworkers reported that forgetfulness, stigma, and lack of disclosure were important barriers among PLHIV in India [30]. Some of these were not important barriers for our population. While important domains have been identified by various authors [31,32], the identification of key barriers and customized enhanced counseling will be more effective for achieving viral suppression in these PLHIV.

Previous studies have also implemented EAC among PLHIV [22,25,33,34]. Ekejiuba and colleagues conducted phone-based EAC among a key population in Nigeria [24]. Their population was predominantly male, aged 20-29 years, and nearly all of them had been on ART for only the past 12 months. They found that VL had reduced in 90.5% of PLHIV after the phone-based intervention. In contrast to their study, our population was older, had been on ART for more than two years, and most of them could not be classified as key populations. Another retrospective study by Diress and coworkers evaluated the role of EAC in VL suppression in Ethiopia [35]. The majority of their study population was aged 30 years or older, with a near-equal distribution of males and females; they were on an efavirenz-based regimen, a high proportion had a CD4 count <200 cells/mm³, and the majority had been on ART for two years or more. This study population was much closer to the population in our study. They found that 66% had suppressed VLs after EAC. Nakaye and colleagues also conducted a similar retrospective study in Uganda [36]. The population in their study was older (aged 30 years or older), predominantly female, on first-line ART, and the majority of them had been on ART for more than one year, with 48% of them for more than five years. Interestingly, they also found a VL suppression of 66% post-EAC [36]. Both these studies had a lower VL suppression post-EAC. Other studies have found viral suppression as low as 31% to as high as 91% after EAC [34,37,38]. A recent meta-analysis reported that 85% were linked to EAC, and VL suppression was 51% [16]. In our analyses, the VL suppression post-EAC was 90%. This was perhaps because we were collecting information on possible reasons for poor adherence and providing barrier-specific/domain-specific counseling along with general EAC.

We found that males, ART duration of more than five years, and baseline CD4 count >350 cells/mm³ were significantly associated with a reduction in VL after EAC sessions. However, third-line ART and barriers related to pill-taking practices were less likely to result in viral suppression. Gender has been identified as one of the predictors of viral suppression after EAC. Unlike our study, Diress and colleagues reported that females were more likely to have viral suppression post-EAC [35]. Kaira and colleagues, on the other hand, found that females were less likely to achieve viral suppression after EAC [39]. Furthermore, they found that PLHIV who had been on ART for less than five years were significantly more likely to be virally suppressed; this was contrary to our population, where those on ART for less than five years were less likely to be virally suppressed. Both of the aforementioned studies were conducted in Africa. Other studies have also identified gender, duration of ART, VL, and ART regimens to be associated with VL suppression post-EAC sessions [34,37]. Though some authors have found the number of EAC sessions to be associated with higher viral suppression after EAC [37], others have not found any association between them [39]. In our study, we did not find any association between the number of EAC sessions and viral suppression during the follow-up period. In a study conducted in an Indian setting (in a city close to our setting), the authors found that females were significantly more likely to achieve viral suppression, and they also did not find any association between barriers to adherence and viral suppression [40]. Both of these findings differed from our own findings, where a specific barrier, pill-taking practices, was associated with significantly lower VL suppression. In our study, the two most important barriers did not differ significantly between genders.

Strengths and limitations

We developed a digital application to document key barriers associated with poor adherence and unsuppressed VLs in PLHIV on ART. This helped us tailor our counseling sessions to address specific barriers. These barriers may differ between the general population and key populations (such as female sex workers and men who have sex with men). This was one of the major strengths of our approach. However, since these analyses were based on programmatic data, we did not have a comparison group, so we were unable to determine if customized EAC versus standard EAC resulted in different outcomes in terms of adherence and/or viral suppression. Furthermore, while the digital app was helpful to document barriers and supported appropriate counseling sessions, we do not wish to attribute a specific causal relation between the follow-up results and the use of this app. Additionally, while we did record barriers during subsequent follow-up visits for those who had multiple EAC sessions, we did not conduct a trajectory analysis to include changes over time. Thus, the analysis should be interpreted cautiously as an association between specific barriers at baseline, and we do not wish to attribute causality to these findings. Information about pill burden among PLHIV due to concomitant opportunistic infections, antituberculosis treatment, and other prophylaxis treatment was not captured in the application and, hence, was not considered in the analysis of poor adherence. In addition, there was attrition due to losses to follow-up and deaths; this may introduce a selection bias if the deaths occurred in those in whom VL was not suppressed.

## Conclusions

The study provides valuable information on key barriers to treatment adherence in PLHIV and the effectiveness of EAC in public health settings in developing countries. The development of a digital app helped us document key barriers and domains in PLHIV with poor adherence. The app can be used in urban (central) as well as peripheral or rural ART centers. The main barriers identified were a lack of adequate knowledge about dosage and side effects, as well as not having a fixed schedule for taking pills. Viral suppression was reported in approximately 90% of PLHIV after EAC sessions. However, VL suppression was less likely in those who did not have a fixed time for taking medicines. This is a practical problem that needs to be addressed by developing treatment plans that consider time spent away from home or in transit. Digital applications such as "Samvaad" can be used as part of service delivery programs in various ART centers for the documentation of barriers to ART adherence and to provide barrier-specific counseling sessions for improving adherence.
